# Supplementary material for: Effect of Probiotic Supplementation on Gut Microbiota in Patients with Major Depressive Disorders: A Systematic Review
Source: Nutrients. 2023 Mar 10;15(6):1351. doi: 10.3390/nu15061351 (PMC10052013; doi:10.3390/nu15061351)
Supplement: Supplementary file 1 [file nutrients-15-01351-s001.zip › nutrients-2270164-supplementary.pdf]

Supplementary Material S1

Table S1. Full search strategy for the various databases

Search strategy for Medline Search

Depression	exp Depressive Disorder/ or exp Mood Disorders/ or exp Depression/ or (depress* or dysthymi* or "affective disorder*" or "depressive disorder" or MDD or suicide).ti,ab.
Gut microbiota	exp Microbiota/ or exp Gastrointestinal Microbiome/ or (microbiota* or gut).ti,ab.
Probiotics	exp Probiotics/ or (probiotic* or lactobacillus or Bifidobacterium).ti,ab.

Search strategy for EMBASE

Depression	'mood disorder'/exp OR 'mood disorder' OR 'depression'/exp OR 'depression' OR depress*:ti,ab OR dysthymi*:ti,ab OR 'affective disorder*':ti,ab OR 'depressive disorder':ti,ab OR mdd:ti,ab OR suicide:ti,ab
Gut microbiota	'intestine flora'/exp OR 'intestine flora' OR microbiota*:ti,ab OR gut:ti,ab
Probiotics	'probiotic agent'/exp OR 'probiotic agent' OR probiotic*:ti,ab OR lactobacillus:ti,ab OR bifidobacterium:ti,ab
Article Filter	‘article’/it

Search strategy for Cochrane Library

#1	MeSH descriptor: [Depression] explode all trees
#2	MeSH descriptor: [Mood Disorders] explode all trees
#3	MeSH descriptor: [Depressive Disorder] explode all trees
#4	((depress* or dysthymi* or "affective disorder*" or "depressive disorder" or MDD or suicid*)):ti,ab,kw
#5	MeSH descriptor: [Microbiota] explode all trees
#6	MeSH descriptor: [Gastrointestinal Microbiome] explode all trees
#7	(microbiota* or gut):ti,ab,kw
#8	MeSH descriptor: [Probiotics] explode all trees
#9	(probiotic* or lactobacillus):ti,ab,kw
#10	#1 or #2 or #3 or #4
#11	#5 or #6 or #7
#12	#8 or #9
#13	#10 and #11 and #12

Supplementary Material S2

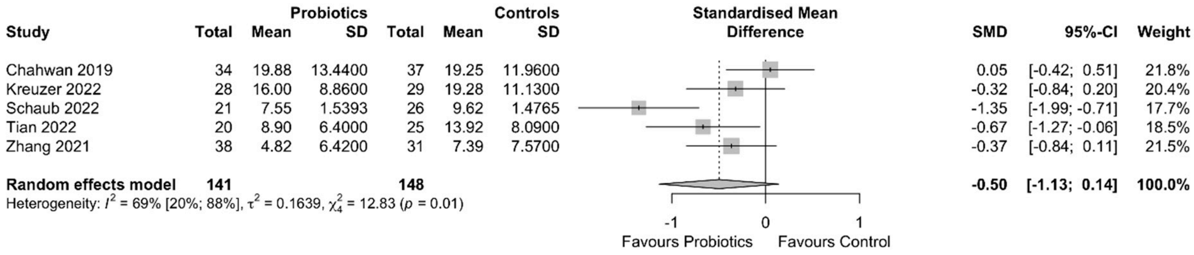

Figure S1. Forest plot showing the standardized mean difference for change in depressive rating scales, post-probiotic intervention
